# Epidemiological characteristics of tuberculosis among migrant populations and key predictors of migratory cases in Zhejiang Province

**DOI:** 10.3389/fpubh.2026.1948462

**Published:** 2026-09-17

**Authors:** Zhengwei Liu, Jiani Miao, Rongrong Qu, Can Chen, Mengsha Chen, Jiaxing Qi, Wenkai Zhou, Huihui Zhang, Mingwu Zhang, Bin Chen, Liqun Fang, Shigui Yang

**Affiliations:** 1 Department of Emergency Medicine, Second Affiliated Hospital, School of Public Health, Zhejiang University School of Medicine, Hangzhou, China; 2 Department of Tuberculosis Control and Prevention, Zhejiang Provincial Center for Disease Control and Prevention, Hangzhou, China; 3 Beijing Institute of Microbiology and Epidemiology, Academy of Military Medical Science, Beijing, China

**Keywords:** boosted regression trees, epidemiological characteristics, migrant populations, spatial heterogeneity, tuberculosis

## Abstract

**Objectives:**

Population mobility poses challenges to tuberculosis (TB) control. As a major destination for internal migrants in China, Zhejiang Province bears a substantial burden of migrant TB cases. This study aimed to characterize TB epidemiology in Zhejiang Province and identify key predictors of migratory TB cases.

**Methods:**

TB surveillance data from the Tuberculosis Information Management System (TBIMS) of Zhejiang Province for 2005–2023 were analyzed using joinpoint regression, age-period-cohort modeling, and spatial analyses to characterize temporal, demographic, and spatial patterns and compare migrant and local TB cases. Boosted regression tree (BRT) modeling was used in a case-case design to identify individual- and municipal-level predictors of migratory status among TB cases and quantify their relative contributions.

**Results:**

The average annual TB incidence was 61.35 per 100,000 population, and migrant TB cases accounted for 34.48% of all reported cases. TB incidence increased during 2005–2007 with an annual percent change (APC) of 2.76%, and subsequently declined during 2007–2023 (APC = −4.12%). The proportion of migrant TB cases increased through 2019 (APC = 5.83%) and then declined sharply during 2019–2023 (APC = −17.62%). Age-period-cohort analysis revealed a bimodal age effect, with peaks at 15–19 and 75–79 years, while cohort effects were elevated among cohorts born from the late 1940s through the 1970s and subsequently declined. Spatial autocorrelation in TB incidence increased from 2010 onward, while local and migrant TB case proportions clustered mainly in Quzhou and Lishui and in Ningbo and Jiaxing, respectively. Gross regional product (GRP) per capita was the leading predictor of migratory TB cases (57.5%), followed by the immigration index (9.0%), city type (6.8%), and tertiary industry proportion of GRP (5.9%), with marked variation in relative contributions across origin regions.

**Conclusions:**

TB incidence declined overall in Zhejiang Province, but migrant TB cases remained epidemiologically important and showed distinct demographic and spatial patterns. GRP per capita was the leading predictor, with additional contributions from population mobility indicators and marked variation across origin regions. These findings support coordinated cross-regional surveillance, continuity of TB care, and strategies adapted to local population structures and mobility patterns.

## Introduction

Tuberculosis (TB) remains a major global public health concern. China accounted for 6.5% of the estimated global incident TB cases in 2024 (1). Despite continued global efforts in TB control, progress remains insufficient to achieve the targets of the World Health Organization (WHO) End TB Strategy (1, 2). Population mobility, including intraprovincial and interprovincial migration, poses additional challenges to TB prevention and control (3). Recent national surveillance research identified substantial intercity mobility among notifiable infectious disease cases, with TB among the diseases with the largest numbers of migratory cases (4).

Rapid urbanization and regional economic disparities have driven large-scale population mobility from less economically developed to more developed regions in China (5). Migrant populations make substantial contributions to economic development. However, large-scale population mobility can reshape contact networks and facilitate the spatial spread of infectious diseases (3, 6–8). Barriers to healthcare access, unstable living conditions, and limited social protection may also complicate timely TB diagnosis and treatment continuity, particularly when care involves multiple administrative regions (9–12).

Zhejiang Province is a major destination for migrant populations in eastern China. During 2013–2017, migrant TB cases accounted for approximately one-third of TB cases in the province (13). This substantial burden makes Zhejiang an important setting for comparing TB patterns between migrant and local TB cases. Previous studies have described the burden and spatial distribution of TB among migrant populations in Zhejiang and other regions of China (3, 14, 15). However, long-term differences in TB patterns between migrant and local TB cases in Zhejiang remain insufficiently characterized. Predictors of migratory TB cases and their variation across regions of origin also remain insufficiently understood (4, 15, 16).

This study aimed to characterize the temporal, demographic, and spatial patterns of TB in Zhejiang Province from 2005 to 2023, with comparisons between migrant and local TB cases. We further identified key predictors of migratory TB cases and assessed heterogeneity across regions of origin. The findings may inform targeted TB surveillance and control strategies for migrant populations.

## Methods

### Data source and study population

This study included all TB cases recorded in the Tuberculosis Information Management System (TBIMS) of Zhejiang Province between 2005 and 2023. The TBIMS is administered by the Zhejiang Provincial Center for Disease Control and Prevention (Zhejiang CDC). TB cases were classified as bacteriologically confirmed pulmonary TB (PTB), clinically diagnosed PTB, or extrapulmonary TB (EPTB) using the diagnostic and classification categories recorded in the TBIMS, according to the national standards in force at the time of notification.

Individual records included sex, age, notification year, occupation, household registration type, household registration code, and current residence code. Direct personal identifiers, including names, identification numbers, and detailed addresses, were removed to protect privacy. Annual resident population data, including population counts by sex, age group, and geographic area, were obtained from the Zhejiang Statistical Yearbooks. Overall reported TB incidence and incidence stratified by sex, age group, and geographic area were calculated using the corresponding annual resident population as the denominator.

Migrant and local TB cases were classified according to the household registration type recorded in the TBIMS (13). Their proportions were calculated using all notified TB cases in the corresponding year, sex, age group, or geographic area as the denominator. Separate population denominators for migrant and local populations were unavailable. Therefore, incidence rates were not calculated separately for these groups, and comparisons were interpreted as case composition rather than population-level risk. Household registration and current residence codes were confirmed at case notification. For the origin-destination and BRT analyses, cases whose household registration and current residence codes differed at the county level were classified as migratory TB cases using an address-based classification, consistent with previous research (13). For the origin-destination analysis, household-registration province was defined as the origin and current-residence city as the destination. These analyses were restricted to 2014–2023, when county-level comparison of the two codes was consistently available (Supplementary Table S2).

### Candidate predictors

Candidate predictors were selected based on previous studies of TB epidemiology, socioeconomic conditions, and population mobility, as well as data availability (3, 4). Individual-level predictors included age, sex, and occupation and were extracted from the TBIMS. Municipal-level predictors included gross regional product (GRP) per capita, secondary industry proportion of GRP, tertiary industry proportion of GRP, hospital beds per 1,000 population, the emigration index, the immigration index, city type, and population density.

For the BRT analyses, municipal-level predictors were assigned according to the city of household registration. GRP per capita, the proportions of secondary and tertiary industries in GRP, hospital beds per 1,000 population, and population density were obtained from the China City Statistical Yearbooks, compiled as annual municipal indicators, and linked to individual TB records by notification year and household-registration city. City type was treated as a fixed structural characteristic. The emigration and immigration indices were derived from daily REAL mobility data from AutoNavi Map (AMAP) for 2019, the earliest year for which historical daily REAL mobility data were publicly available and the last complete pre-COVID-19 year. Daily outflow and inflow values were averaged across the year to obtain city-level indices and assigned by household-registration city as fixed reference-period measures of intercity population mobility (4).

### Epidemiological analysis

Joinpoint regression was used to examine temporal trends in TB incidence and the proportion of migrant TB cases in Zhejiang Province from 2005 to 2023. This method identifies points at which temporal trends change significantly and quantifies each segment using the annual percent change (APC) (17). Analyses were performed using the Joinpoint Regression Program, version 5.3.0.

An age-period-cohort model was used to estimate age, period, and cohort effects on reported TB incidence. The analysis used the latest three complete 5-year periods (2009–2023) within the 2005–2023 study period. Age was categorized into 19 5-year groups ranging from 0–4 to 90–94 years. The open-ended ≥95-year group was excluded. Age-period-specific case counts were modeled using Poisson log-linear regression with log population as an offset (18). The intrinsic estimator (IE) was used to address the exact linear dependence among age, period, and cohort (18, 19). Separate models were fitted for the overall population, males, and females, with centered effects expressed as relative risks (RRs), where RR = 1 represents the average effect. Residual deviance and Pearson dispersion were assessed, and 95% confidence intervals (CIs) were based on Pearson-scaled standard errors to account for overdispersion (20). Robustness was assessed by restricting the age range to 0–84 years, using 2005-2019 as an alternative analysis period, and comparing observed age-specific incidence across the three primary periods.

Unsupervised hierarchical clustering was performed across the 11 prefecture-level cities to identify distinct epidemiological patterns in TB incidence and the distribution of migrant and local TB cases. Clustering results were visualized using the pheatmap package, version 1.0.12, in R version 4.3.3. County-level spatial autocorrelation was assessed using global Moran's I with row-standardized inverse-distance spatial weights and a fixed distance threshold (21, 22). Getis-Ord Gi^*^ statistics were calculated using binary spatial weights within the same distance threshold to identify hot spots and cold spots at the 90%, 95%, and 99% confidence levels, with the 90% level considered exploratory (23). Benjamini-Hochberg false discovery rate (FDR) correction was applied to account for multiple local tests (24), and empirical Bayes smoothing of TB incidence was used to assess potential rate instability in smaller populations (25). Spatial analyses were conducted using R version 4.3.3 and ArcGIS version 10.8.2.

Continuous variables were summarized as means and standard deviations, and categorical variables as numbers and percentages. Group comparisons used Fisher's exact test, analysis of variance, the chi-square test, or the independent-samples *t* test, as appropriate. Agreement between household registration type and the address-based classification was assessed using overall agreement and Cohen's κ. All tests were two-sided at α = 0.05.

### Boosted regression tree modeling

Within a case-case study design, a boosted regression tree (BRT) model was developed for the overall analysis, with subgroup analyses conducted for intraprovincial and leading interprovincial origin groups (26). Migratory TB cases constituted the study group, and cases with concordant household registration and current residence constituted the comparison group. The BRT was used to identify key predictors distinguishing migratory TB cases from comparison cases and quantify their relative contributions. For the overall analysis, repeated stratified subsampling without replacement was performed 50 times. Each sample comprised 10% of eligible records within strata defined by notification year and case group and was subsequently divided into training and testing sets at a ratio of 70:30 within the same strata. Subgroup analyses sampled five comparison cases per migratory case, matched by notification year. Temporal and geographic validation assessed model transportability across periods and origin cities, using 2014–2019 for training and 2020–2023 for testing and five-fold whole-origin-city validation, respectively. Sensitivity analyses examined migrant classification using a stricter definition, AMAP temporal alignment by restricting the analysis to 2019 and excluding the AMAP indices, and potential COVID-19 influence by restricting the analysis to 2014–2019.

BRT was selected for its ability to capture nonlinear relationships and interactions among predictors. Tree complexity, learning rate, and bag fraction were set to 4, 0.005, and 0.75, respectively, following prior studies (27, 28). The optimal number of trees was selected by five-fold cross-validation within the training data. Model discrimination was evaluated using the area under the receiver operating characteristic (ROC) curve (AUC). Across the 50 repeated analyses, AUCs and relative contributions were summarized as medians with 95% intervals. Relative contributions quantified predictive importance within the case-case comparison rather than adjusted or causal effects, and fitted functions characterized model-predicted relationships. All 11 prespecified predictors were included in the models. The 2% contribution threshold was used only for presentation. BRT analyses were performed using the dismo package, version 1.3–5, and the gbm package, version 2.1.8.1, in R version 4.3.3. The pROC package, version 1.18.4, was used to calculate ROC curves and AUC values.

## Results

### Epidemiological and demographic characteristics

Between 2005 and 2023, 619,466 TB cases were reported in Zhejiang Province. Household registration type was available for 619,464 cases, of which 213,605 (34.48%) were migrant TB cases. Overall agreement between household registration type and the address-based classification was 86.73% (Cohen's κ = 0.711; Supplementary Table S3). The average annual TB incidence was 61.35 per 100,000 population. Table 1 presents the sociodemographic characteristics of migrant and local TB cases. Males accounted for 69.5% of local TB cases and 66.8% of migrant TB cases. Migrant TB cases were younger than local TB cases (36.0 ± 15.2 years vs. 51.4 ± 19.6 years) and had higher proportions of cases aged 18–34 years (52.8% vs. 22.6%) and migrant workers (37.3% vs. 2.7%). TB incidence increased during 2005–2007 (APC = 2.76%) and subsequently declined during 2007–2023 (APC = −4.12%) (Supplementary Figure S1). The proportion of migrant TB cases increased through 2019 (APC = 5.83%) and then declined sharply during 2019–2023 (APC = −17.62%) (Supplementary Figure S2).

**Table 1:** Sociodemographic characteristics of migrant and local TB cases in Zhejiang, 2005–2023.

Name	Levels	Local TB cases (*N* = 405,859)	Migrant TB cases (*N* = 213,605)	*P* value
Sex	Female	123,876 (30.5%)	70,949 (33.2%)	<0.001
	Male	281,983 (69.5%)	142,656 (66.8%)	
Age	Mean ± SD	51.4 ± 19.6	36.0 ± 15.2	<0.001
Age group	0–17	9,443 (2.3%)	6,619 (3.1%)	<0.001
	18–34	91,556 (22.6%)	112,841 (52.8%)	
	35–59	145,451 (35.8%)	74,343 (34.8%)	
	≥60	159,409 (39.3%)	19,802 (9.3%)	
Occupation	Others	13,800 (3.4%)	9,607 (4.5%)	<0.001
	Preschool children	387 (0.1%)	259 (0.1%)	
	Students	17,151 (4.2%)	6,897 (3.2%)	
	Teachers	2,265 (0.6%)	592 (0.3%)	
	Service workers	12,125 (3.0%)	10,725 (5.0%)	
	Healthcare workers	2,406 (0.6%)	604 (0.3%)	
	Workers	32,364 (8.0%)	30,076 (14.1%)	
	Migrant workers	11,044 (2.7%)	79,765 (37.3%)	
	Agricultural and fishery workers	249,691 (61.5%)	42,858 (20.1%)	
	Sailors and long-distance drivers	410 (0.1%)	204 (0.1%)	
	Office workers	6,899 (1.7%)	3,537 (1.7%)	
	Retirees	22,993 (5.7%)	4,817 (2.3%)	
	Household and unemployed	34,324 (8.5%)	23,664 (11.1%)	

Data are presented as *n* (%) unless otherwise indicated. Two cases with missing household registration type were excluded. SD, standard deviation.

Figures 1A, B show age- and sex-specific patterns in TB incidence and the proportion of migrant TB cases, respectively. Figures 1C–E present the age, period, and cohort effects on TB incidence. The age effect showed a bimodal pattern, with the first and highest peak at 15–19 years (RR = 4.91, 95% CI: 4.00–6.01). It subsequently declined before increasing again to a second peak at 75–79 years (RR = 2.46, 95% CI: 2.13–2.86). Similar patterns were observed in both sexes, although the second peak occurred at 75–79 years in males and 80–84 years in females. Period effects declined progressively from 2009 to 2023. Cohort effects increased across earlier birth cohorts and peaked among those born during 1954–1958 (RR = 2.74, 95% CI: 2.39–3.14). The effects subsequently declined and fell below the average cohort-effect level in more recent birth cohorts. All primary and sensitivity models converged, although model diagnostics indicated overdispersion (Supplementary Table S4). The overall age pattern and declining period effects were broadly consistent across both sensitivity analyses, while the broader cohort pattern was retained (Supplementary Figure S4). Observed age-specific incidence across the three primary periods showed a similar bimodal pattern (Supplementary Figure S3).

**Figure 1:**
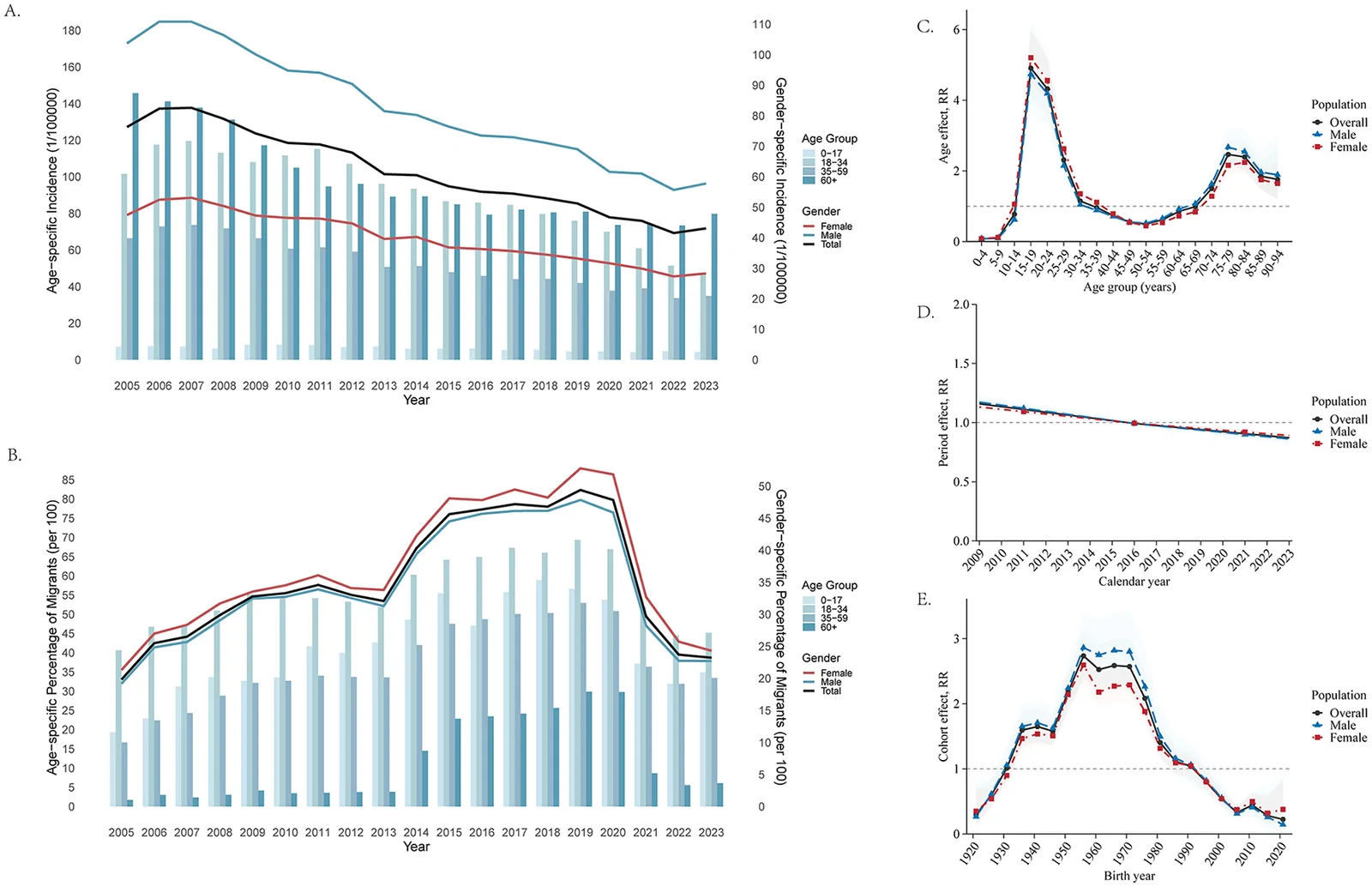
Population distribution characteristics of TB in Zhejiang Province, 2005–2023. **(A)** Age- and sex-specific reported TB incidence, 2005–2023. **(B)** Age- and sex-specific proportions of migrant TB cases, 2005–2023. **(C–E)** Age, period, and cohort effects on reported TB incidence, 2009–2023. Shaded areas indicate 95% CIs.

### Spatiotemporal heterogeneity of TB

Figure 2 presents the spatiotemporal distribution of TB incidence and the proportions of local and migrant TB cases across Zhejiang Province from 2005 to 2023. City-level TB incidence showed marked spatial heterogeneity across the study period. Figures 2A–C present the hierarchical clustering of TB incidence and the proportions of migrant and local TB cases. Based on TB incidence, Hangzhou, Jinhua, Ningbo, and Quzhou formed the higher-incidence cluster, whereas the remaining seven cities formed the lower-incidence cluster. Within the higher-incidence cluster, Jinhua and Quzhou showed relatively higher proportions of local TB cases, whereas Hangzhou and Ningbo showed relatively higher proportions of migrant TB cases.

**Figure 2:**
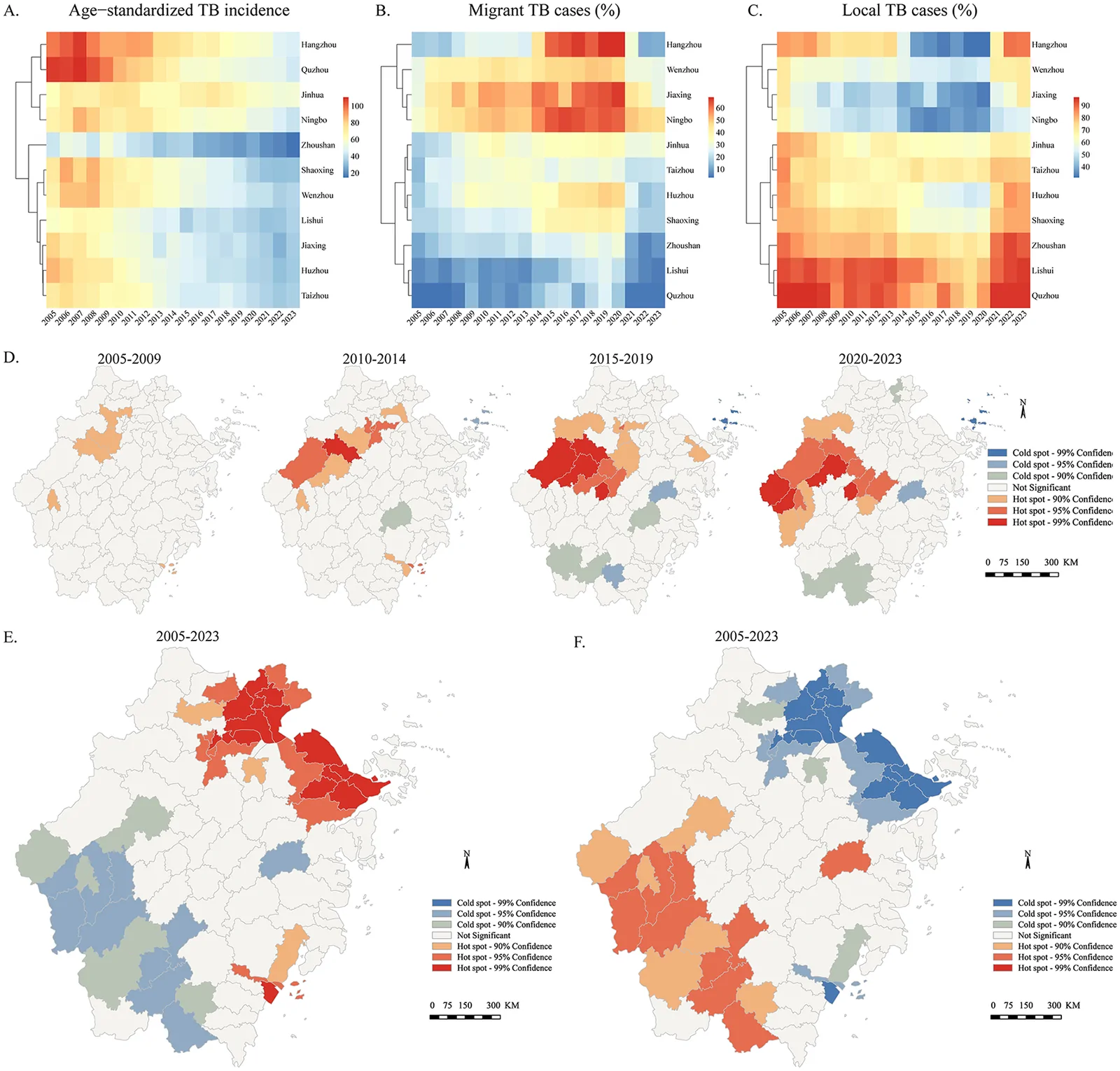
Spatiotemporal distribution and clustering of reported TB incidence and the proportions of migrant and local TB cases in Zhejiang Province, 2005–2023. **(A–C)** Unsupervised hierarchical clustering of annual city-level age-standardized reported TB incidence **(A)**, migrant TB case proportion **(B)**, and local TB case proportion **(C)**. Rows represent cities, columns represent years, and cell color indicates the value of each epidemiological indicator. Cities were hierarchically clustered, while years are shown in chronological order. **(D)** County-level Getis-Ord Gi* hot and cold spots of reported TB incidence for 2005–2009, 2010–2014, 2015–2019, and 2020–2023. **(E, F)** County-level Getis-Ord Gi* hot and cold spots in the proportions of migrant **(E)** and local **(F)** TB cases for the overall period 2005–2023. Maps display the 90%, 95%, and 99% confidence levels; the 90% level was considered exploratory. TB, tuberculosis.

Geospatial analysis showed changing patterns of spatial autocorrelation in TB incidence over time (Figure 2D). Global Moran's I showed no significant spatial autocorrelation during 2005–2009, followed by increasingly positive spatial autocorrelation from 2010 onward. The proportions of migrant and local TB cases showed persistent positive spatial autocorrelation across all periods (Supplementary Table S5). Hot spots in TB incidence expanded from the Hangzhou area toward Jinhua and Quzhou over time. Hot spots in the proportion of migrant TB cases were consistently observed in Ningbo and Jiaxing, with an additional cluster in Hangzhou during 2015–2019 (Figure 2E; Supplementary Figure S5). Hot spots in the proportion of local TB cases remained concentrated in Quzhou and Lishui, with intermittent clustering in Taizhou and parts of Hangzhou (Figure 2F; Supplementary Figure S5). Sensitivity analyses showed similar spatial incidence patterns after empirical Bayes smoothing (Supplementary Table S6). After FDR correction, no incidence-based hot or cold spots remained significant, whereas several clusters based on migrant and local case proportions remained significant (Supplementary Figure S6).

Figure 3 presents the origin-destination patterns of interprovincial migratory TB cases in Zhejiang Province during 2014–2023. Among 67,916 cases, Guizhou was the leading origin province, accounting for 16,832 cases (24.8%), followed by Sichuan and Anhui (Figure 3A; Supplementary Table S7). Ningbo received the largest number of cases, followed by Wenzhou and Hangzhou (Figure 3B). The 10 leading origin provinces accounted for 91.1% of all interprovincial cases, with destination distributions varying across origin provinces (Figure 3C). Annual case counts were generally lower after 2019 across most leading origin provinces, although temporal patterns varied by province (Figure 3D).

**Figure 3:**
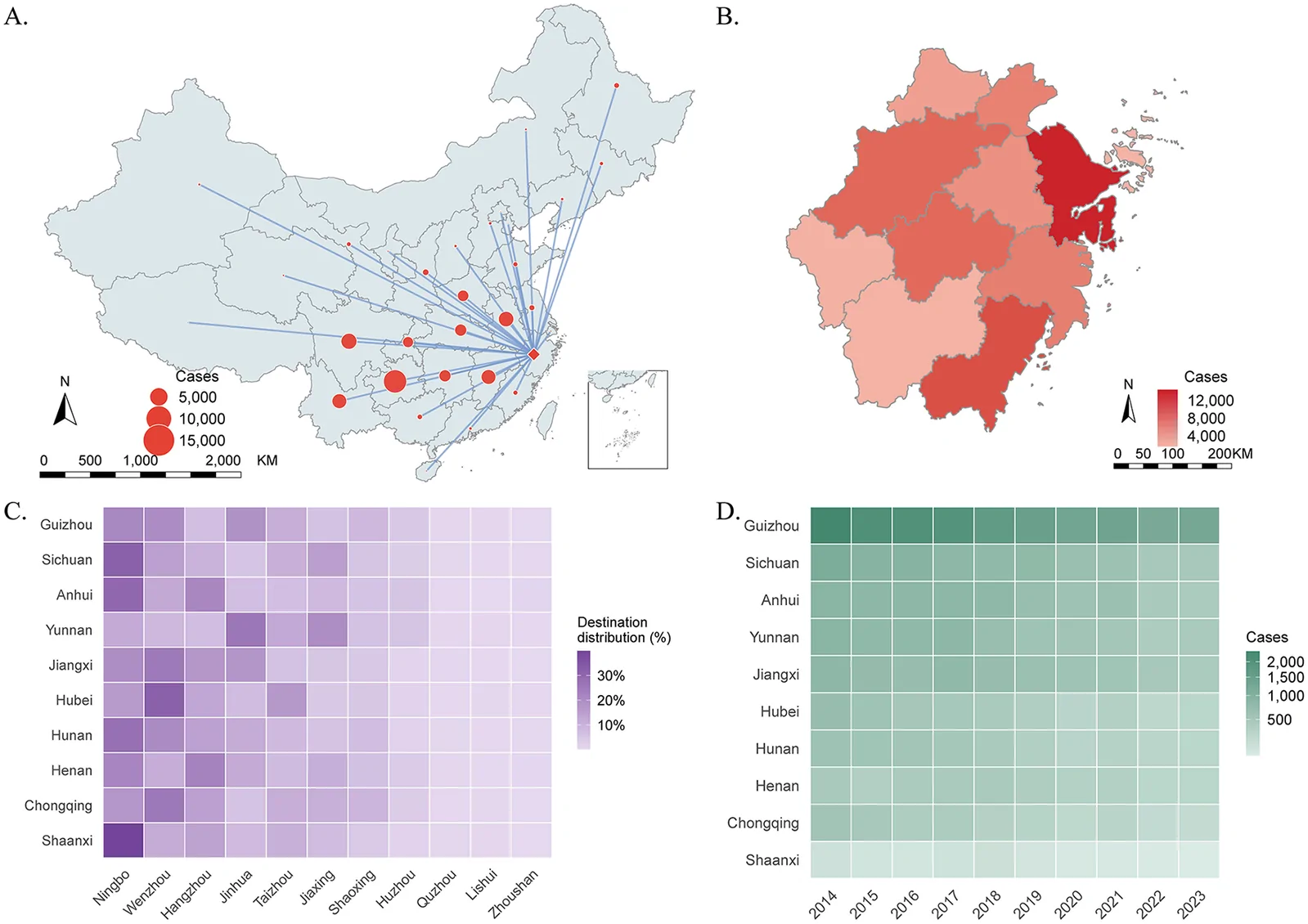
Origin-destination patterns of interprovincial migratory TB cases in Zhejiang Province, 2014-2023. **(A)** Geographic origins and flows of interprovincial migratory TB cases from household-registration provinces to Zhejiang Province. Circle size represents the number of cases. **(B)** Destination distribution of cases across current-residence cities in Zhejiang Province. Color intensity represents the number of cases. **(C)** Destination distribution among the 10 leading origin provinces. Color intensity represents the percentage of cases from each origin province distributed across destination cities. **(D)** Annual case counts for the 10 leading origin provinces during 2014–2023. Color intensity represents the number of cases. Household-registration province was defined as the origin and current-residence city as the destination. TB, tuberculosis.

### Key predictors of migratory TB cases

In the overall BRT analysis, GRP per capita had the largest median relative contribution at 57.5% (95% interval: 54.5%−59.5%), followed by the immigration index at 9.0% (7.3%−12.3%), city type at 6.8% (6.0%−7.8%), and tertiary industry proportion of GRP at 5.9% (4.7%−7.4%) (Table 2). The median training and testing AUCs were 0.973 (0.970–0.976) and 0.967 (0.961–0.971), respectively (Figure 4B). Temporal validation yielded an AUC of 0.924, while the median AUC across the five geographic-validation folds was 0.841 (range: 0.740–0.969). GRP per capita remained the leading predictor in temporal validation (64.9%) and geographic validation (median, 53.5%; range, 49.9%−69.1%) (Supplementary Table S8). Across the sensitivity analyses, testing AUCs ranged from 0.956 to 0.972, and GRP per capita remained the leading predictor, although the relative contributions of several secondary predictors varied (Supplementary Table S9).

**Table 2:** Relative contributions of predictors in the overall, intraprovincial, and origin-specific boosted regression tree analyses, Zhejiang Province, 2014–2023.

Variable	Overall *n* = 75,017	Intra-provincial *n* = 9,064	Anhui *n* = 6,908	Hubei *n* = 4,006	Guizhou *n* = 16,423	Hunan *n* = 4,109	Jiangxi *n* = 6,296	Sichuan *n* = 7,558	Yunnan *n* = 6,488	Henan *n* = 3,786
GRP per capita	57.5 (54.5–59.5)	6.0 (3.4–10.5)	61.3 (57.5–65.2)	41.2 (28.6–48.7)	65.9 (62.6–69.2)	69.5 (63.0–75.7)	43.8 (35.0–52.5)	27.1 (12.4–45.9)	56.2 (32.0–73.9)	67.7 (61.1–72.6)
Tertiary industry proportion	5.9 (4.7–7.4)	2.7 (1.6–4.9)	7.4 (4.5–9.9)	12.0 (4.7–16.6)	9.2 (6.6–11.6)	6.1 (3.7–8.3)	8.2 (3.8–14.7)	10.7 (5.7–30.3)		3.2 (1.1–5.9)
Emigration index	4.3 (3.5–5.5)	5.8 (2.3–10.4)	7.6 (4.6–11.6)	8.8 (5.1–23.0)			2.4 (1.2–4.8)	38.2 (20.4–51.4)	13.9 (2.7–35.6)	
Immigration index	9.0 (7.3–12.3)	3.0 (0.7–6.5)	19.3 (12.9–24.9)	20.0 (13.8–32.7)	4.9 (3.6–6.6)	12.9 (9.8–16.6)	24.1 (17.8–29.5)	12.7 (6.1–19.2)	11.2 (5.9–48.1)	5.8 (2.8–11.9)
Population density	4.2 (3.0–5.4)	6.3 (3.6–9.9)		6.7 (4.4–10.6)			13.9 (9.9–21.7)	3.6 (0.5–7.8)	7.0 (3.6–11.6)	12.7 (7.5–16.3)
City type	6.8 (6.0–7.8)			3.7 (0.6–5.1)	3.5 (2.7–4.9)			7.4 (5.9–9.3)	3.6 (1.9–5.5)	2.4 (0.7–4.7)
Age	3.3 (2.8–3.9)	23.8 (18.3–31.1)								
Occupation	4.5 (3.7–5.3)	41.7 (33.2–48.3)		3.8 (1.5–7.0)						
Hospital beds	3.5 (2.9–4.5)	4.0 (1.9–7.0)				4.1 (1.5–7.9)				2.7 (1.3–7.8)
Secondary industry proportion		3.2 (1.5–6.0)			9.4 (7.7–11.5)					3.2 (0.9–6.7)
Sex										

Values are median relative contributions (%) with 95% intervals across 50 repeated analyses. *n* denotes eligible migratory TB cases. Subgroup models sampled five notification-year-matched comparison cases per migratory case. Origin-specific models included leading eligible provinces by case count until ≥90% of eligible interprovincial cases were covered; eligibility required ≥2 household-registration cities. Values <2% are omitted. GRP, gross regional product.

**Figure 4:**
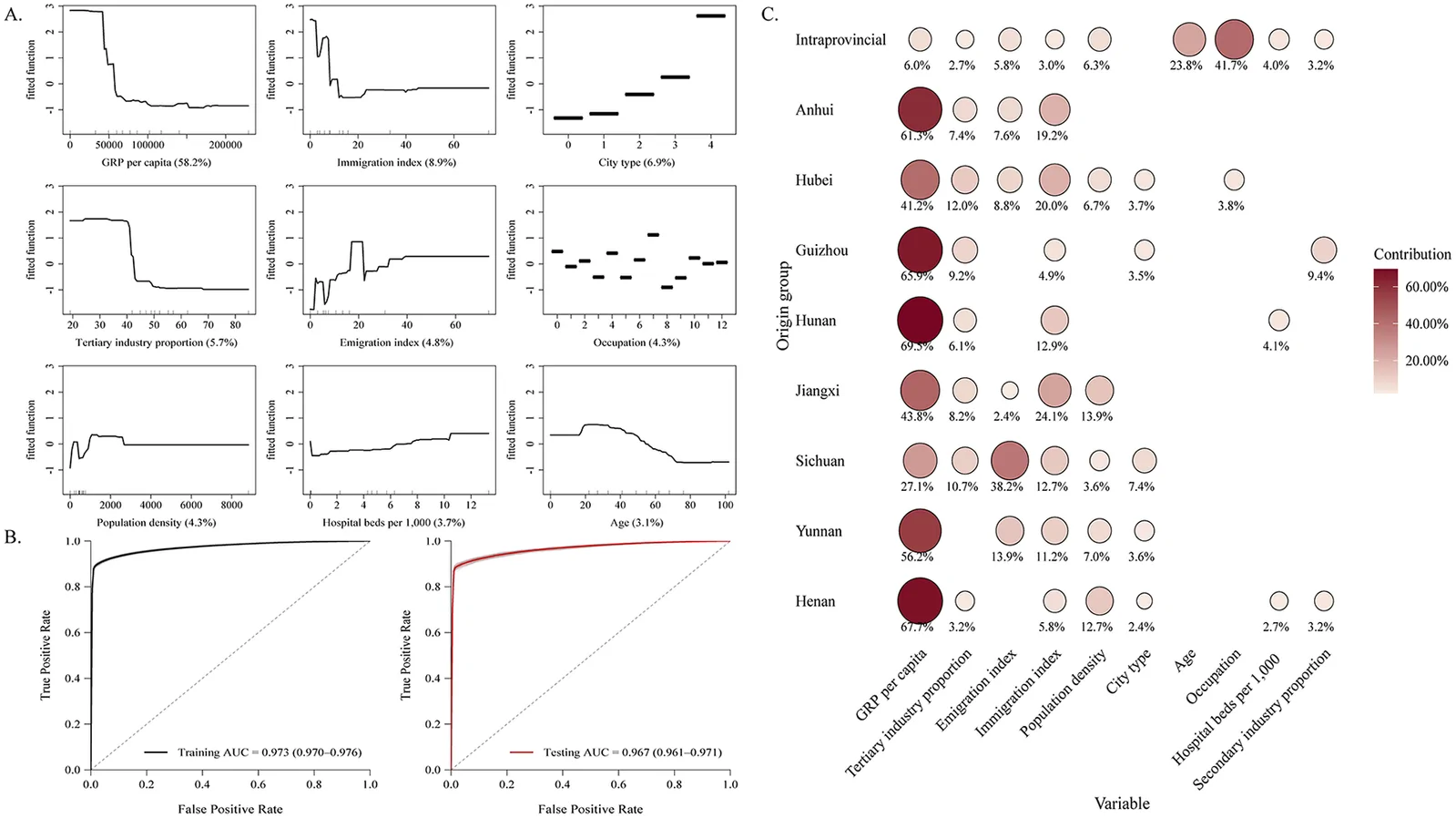
Predictors of migratory TB cases in Zhejiang Province: overall and subgroup boosted regression tree analyses. **(A)** Fitted functions for the leading predictors from the overall BRT model fitted to the complete analytical dataset. Percentages in parentheses indicate relative contributions from this model. **(B)** Receiver operating characteristic curves for the overall BRT model. Across 50 repeated analyses, training and testing AUCs are summarized as medians with 95% intervals, and shaded bands show pointwise 95% empirical intervals for the ROC curves (2.5th−97.5th percentiles). **(C)** Median relative contributions of predictors in the intraprovincial and origin-specific subgroup analyses. Predictors with median relative contributions <2% are omitted for presentation. AUC, area under the receiver operating characteristic curve; BRT, boosted regression tree; GRP, gross regional product.

The fitted functions showed nonlinear relationships between the leading predictors and migratory TB case status (Figure 4A). Several predictors, including GRP per capita and tertiary industry proportion of GRP, showed marked threshold-like changes, while others displayed stepwise or non-monotonic patterns across their observed ranges. The relative contributions of predictors varied substantially across origin groups (Figure 4C and Table 2). In the intraprovincial model, occupation (41.7%) and age (23.8%) had the largest contributions. Among the eight interprovincial origin groups, GRP per capita was the leading predictor in seven, with contributions ranging from 41.2% to 69.5%. Sichuan showed a different profile, with the emigration index contributing most strongly (38.2%), followed by GRP per capita (27.1%).

## Discussion

This study extends previous research on migrant TB in Zhejiang Province by integrating long-term epidemiological trends with cross-regional mobility patterns of TB cases (13, 15). The overall decline in TB incidence coexisted with persistent challenges posed by population mobility. Migrant TB cases remained concentrated among young working-age adults, whereas the local TB burden was more prominent among older adults. Moreover, interprovincial migratory TB cases were concentrated in several major origin provinces and showed differing destination distributions, while GRP per capita was the leading predictor in the overall BRT model. These findings demonstrate substantial demographic, spatial, and origin-specific heterogeneity in TB patterns associated with population mobility.

The sustained decline in TB incidence after 2007 is consistent with broader progress in TB control in China and may reflect cumulative improvements in case detection, standardized treatment, and public health management (29). In contrast, the sharp decline in the proportion of migrant TB cases after 2019 may reflect changes in both population mobility and TB service delivery. COVID-19-related restrictions may have reduced population movement, but the pandemic also disrupted TB diagnosis, notification, and follow-up services (30, 31). The abrupt reversal after 2019 may therefore represent a pandemic-related disruption of mobility and case detection rather than a continuation of the pre-2019 trend. More broadly, the post-2019 temporal and spatial patterns may partly reflect pandemic-related disruptions rather than underlying long-term changes, making pre-2020 patterns a more appropriate reference for longer-term epidemiology.

The bimodal age pattern points to two distinct age-related priorities for TB prevention and surveillance, consistent with previous observations (18, 32). The adolescent peak may be related to prolonged close contact in schools, particularly in boarding and senior high school settings, which may facilitate TB transmission (33, 34). The later peak among older adults may reflect the combined effects of cumulative exposure, reactivation of latent TB infection, immunosenescence, and chronic comorbidities (35, 36). These findings support age-specific surveillance and timely diagnosis in school settings and among older adults rather than a uniform approach across age groups. Routine surveillance data cannot, however, distinguish the relative contributions of recent transmission and reactivation to these age-specific patterns. The elevated cohort effects among individuals born from the late 1940s through the 1970s may reflect greater infection pressure and poorer living and healthcare conditions earlier in life (37). The subsequent decline in later cohorts is compatible with long-term improvements in nutrition, healthcare access, and TB control (37).

The contrasting spatial patterns of migrant and local TB cases likely reflect different demographic and socioeconomic contexts within Zhejiang. Manufacturing cities with large migrant populations attract working-age migrants through concentrated employment opportunities and frequent cross-regional labor mobility. In western Zhejiang, the higher proportion of local TB cases may partly reflect the older and more rural profile observed among local TB cases. Previous studies across China have similarly shown that internal migration is associated with the spatial distribution of TB and regional heterogeneity of TB burden (3, 13, 38, 39). Cities with large migrant populations may require accessible diagnostic and management services, workplace and community health education, and continuity of care across administrative areas. In western areas with a higher proportion of local TB cases, routine TB surveillance and case management should place greater emphasis on older and rural populations.

The origin-destination analysis places interprovincial TB case flows in Zhejiang within the broader context of domestic labor migration and regional inequality. National studies have shown that TB spatial patterns overlap with population outflow and that per capita GDP, emigration, immigration, and origin-region TB burden are associated with TB case flows (3, 16, 39). In the present study, interprovincial cases were concentrated in several major origin provinces and destination distributions differed across origin provinces. However, annual origin-specific migration denominators to Zhejiang were unavailable, so these patterns should be interpreted descriptively rather than as evidence of origin-specific TB incidence or population-level risk. This analysis also cannot determine where infection or disease progression occurred, as notified cases may reflect infection before migration, reactivation after migration, recent transmission at the destination, or movement for diagnosis and care. These findings support coordinated surveillance and case management across origin and destination regions, particularly between major origin provinces and destination cities receiving large numbers of cases (16).

The study used two related but distinct approaches to classify migrant TB cases. Household registration type supported the long-term comparisons between migrant and local TB cases, whereas the address-based classification defined migratory TB cases for the origin-destination and BRT analyses. This distinction avoids conflating differences between household registration and current residence with geographic changes occurring during TB diagnosis and management. Within this case-case design, the BRT characterized the individual and municipal features distinguishing migratory TB cases from comparison cases with concordant household registration and current residence. Relative contributions reflect predictive importance within this comparison rather than adjusted or causal effects and do not estimate TB risk in the underlying population. GRP per capita provided the largest predictive contribution in the overall model. Because municipal predictors were linked to the household-registration city, the predominance of GRP per capita suggests that the socioeconomic context of origin regions provided substantial predictive information rather than indicating a direct epidemiological effect of GRP itself. Regional socioeconomic conditions are closely related to employment opportunities and labor migration. The markedly higher proportion of migrant workers among migrant TB cases (Table 1) is consistent with the prominent role of labor mobility in this study population. A national TB flow model found that higher per capita GDP in origin regions was associated with lower TB outflows, whereas higher GDP in destination regions was associated with greater inflows (16). The contrasting fitted relationships of the emigration and immigration indices suggest that population outflow and inflow contexts contributed differently to model predictions. Tertiary industry proportion of GRP may reflect service-sector employment, urban development, and demand for mobile labor (3, 40). Population density may capture the concentration of workers, intensity of population movement, and crowded living or working environments (40, 41). The fitted functions indicated that these relationships varied across the observed predictor ranges rather than following simple linear patterns. Model discrimination remained high in temporal validation but was lower in geographic validation, suggesting more limited transportability to previously unseen origin cities.

Origin-specific models revealed marked heterogeneity in relative contributions. In the intraprovincial model, occupation and age contributed most strongly, whereas GRP per capita was the leading predictor in seven of the eight interprovincial origin groups. Sichuan differed from this general pattern, with the emigration index providing the largest contribution. This heterogeneity may reflect differences in socioeconomic conditions, labor mobility, and population structure (16). These differences support a common cross-regional TB control framework with implementation adapted to local migration patterns and service capacity. Regions connected by large labor flows require timely information sharing and continuity of care, while major employment destinations require accessible diagnostic and management services for mobile workers (10–12).

Several limitations should be acknowledged. First, the study was limited to notified TB cases. Population denominators were unavailable separately for migrant and local populations, so comparisons between these groups were based on case proportions rather than incidence rates. Second, the address-based classification did not capture migration timing or duration of residence and therefore could not distinguish recent migrants from long-term residents. It also could not determine where TB infection occurred. Third, assigning municipal-level predictors to individual cases may have introduced ecological bias, and the fixed 2019 AMAP indices may not fully represent population mobility in other years. Their relative contributions may also be sensitive to temporal specification. Information on income, housing, and healthcare access was unavailable. Finally, geographic validation showed lower discrimination in previously unseen origin cities, while the smaller samples in origin-specific models may have increased sensitivity to sample composition.

## Conclusion

TB incidence declined overall in Zhejiang Province, while migrant TB cases accounted for more than one-third of all reported cases and were concentrated among younger working-age adults. TB incidence showed bimodal age patterns and marked cohort variation, while migrant and local TB cases differed demographically and spatially. Interprovincial migratory TB cases were concentrated in several major origin provinces and showed differing destination distributions. In the BRT case-case analysis, GRP per capita was the leading predictor, with marked variation in relative contributions across origin regions. These findings support coordinated cross-regional surveillance, continuity of TB care, and strategies adapted to local population structures and mobility patterns.

## Data Availability

The data analyzed in this study is subject to the following licenses/restrictions: The datasets analyzed in this study are not publicly available because they consist of individual-level tuberculosis surveillance records and are subject to privacy, confidentiality, and institutional data-use restrictions. Requests to access the datasets should be directed to the Zhejiang Provincial Center for Disease Control and Prevention and are subject to approval by the relevant institutional authorities. Requests to access these datasets should be directed to Zhengwei Liu, zhwliu@cdc.zj.cn.
