# Supplementary material for: Epidemiological characteristics of tuberculosis among migrant populations and key predictors of migratory cases in Zhejiang Province
Source: Front Public Health. 2026 Sep 17;14:1948462. doi: 10.3389/fpubh.2026.1948462 (PMC13627407; doi:10.3389/fpubh.2026.1948462)
Supplement: Supplementary file 1 [file Data_Sheet_1.docx]

Supplementary Material

Supplement to: Epidemiological Characteristics of Tuberculosis Among Migrant Populations and Key Predictors of Migratory Cases in Zhejiang Province

**Contents**

Supplementary Tables	S1-S9

Supplementary Figures	S1-S6

1 **Supplementary Tables**

Table S1. Summary of Tuberculosis Surveillance Data in Zhejiang Province, 2005-2023.

| Name | Levels | Reported Cases | Incident Cases | Fatal Cases |
| --- | --- | --- | --- | --- |
| Sex | Female | 194825 (31.5%) | 180389 (32.0%) | 403 (18.7%) |
|  | Male | 424641 (68.5%) | 382945 (68.0%) | 1756 (81.3%) |
| Age | Mean ± SD | 46.1 ± 19.6 | 45.5 ± 19.6 | 69.2 ± 16.0 |
| Age Group | 0-17 | 15983 (2.6%) | 15589 (2.8%) | 4 (0.2%) |
|  | 18-34 | 204280 (33.0%) | 192104 (34.1%) | 113 (5.2%) |
|  | 35-59 | 219794 (35.5%) | 197777 (35.1%) | 338 (15.7%) |
|  | ≥60 | 179209 (28.9%) | 157680 (28.0%) | 1703 (78.9%) |
| Occupation | Others | 22937 (3.8%) | 20936 (3.8%) | 40 (1.9%) |
|  | Preschool Children | 594 (0.1%) | 572 (0.1%) | 2 (0.1%) |
|  | Students | 23659 (3.9%) | 23110 (4.2%) | 4 (0.2%) |
|  | Teachers | 2690 (0.4%) | 2547 (0.5%) | 2 (0.1%) |
|  | Service Workers | 22271 (3.7%) | 20978 (3.8%) | 16 (0.8%) |
|  | Healthcare Workers | 2883 (0.5%) | 2789 (0.5%) | 0 (0.0%) |
|  | Workers | 60943 (10.0%) | 56537 (10.2%) | 52 (2.4%) |
|  | Migrant Workers | 89346 (14.7%) | 81274 (14.7%) | 65 (3.0%) |
|  | Agricultural and Fishery Workers | 289434 (47.5%) | 259620 (46.9%) | 1500 (70.3%) |
|  | Sailors and Long-Distance Drivers | 556 (0.1%) | 506 (0.1%) | 0 (0.0%) |
|  | Office Workers | 10175 (1.7%) | 9674 (1.7%) | 4 (0.2%) |
|  | Retirees | 27433 (4.5%) | 24328 (4.4%) | 269 (12.6%) |
|  | Household and Unemployed | 56389 (9.3%) | 51201 (9.2%) | 179 (8.4%) |
| Household Registration Type | Local | 405859 (65.5%) | 367078 (65.2%) | 1887 (87.4%) |
|  | Intracity Mobility | 139884 (22.6%) | 127839 (22.7%) | 208 (9.6%) |
|  | Intraprovincial Mobility | 7513 (1.2%) | 7133 (1.3%) | 18 (0.8%) |
|  | Interprovincial Mobility | 65562 (10.6%) | 60687 (10.8%) | 46 (2.1%) |
|  | HKMT | 34 (0.0%) | 30 (0.0%) | 0 (0.0%) |
|  | Foreign | 612 (0.1%) | 565 (0.1%) | 0 (0.0%) |

Data are presented as n (%) unless otherwise indicated. Percentages were calculated among cases with non-missing information for each variable. Totals therefore varied because of missing data. For comparisons between migrant and local TB cases, all non-local household registration categories were classified as migrant. SD, standard deviation. HKMT, Hong Kong, Macao, and Taiwan.

Table S2. Annual completeness of geographic codes used for address-based classification among notified tuberculosis cases, 2005-2023.

| Year | Total cases | Household registration code | | Current residence code | | County-level comparison available, n (%) |
| --- | --- | --- | --- | --- | --- | --- |
|  |  | missing, n (%) | incomplete, n (%) | missing, n (%) | incomplete, n (%) |  |
| 2005 | 39247 | 1107 (2.82%) | 5540 (14.12%) | 8 (0.02%) | 0 (0.00%) | 32592 (83.04%) |
| 2006 | 42284 | 298 (0.70%) | 6815 (16.12%) | 0 (0.00%) | 0 (0.00%) | 35171 (83.18%) |
| 2007 | 42913 | 1095 (2.55%) | 5817 (13.56%) | 0 (0.00%) | 0 (0.00%) | 36001 (83.89%) |
| 2008 | 41033 | 295 (0.72%) | 6907 (16.83%) | 1 (0.00%) | 0 (0.00%) | 33830 (82.45%) |
| 2009 | 38457 | 6114 (15.90%) | 7553 (19.64%) | 3 (0.01%) | 0 (0.00%) | 24789 (64.46%) |
| 2010 | 36870 | 24097 (65.36%) | 5653 (15.33%) | 50 (0.14%) | 0 (0.00%) | 7112 (19.29%) |
| 2011 | 36623 | 23806 (65.00%) | 5022 (13.71%) | 9 (0.02%) | 0 (0.00%) | 7793 (21.28%) |
| 2012 | 35662 | 23576 (66.11%) | 4890 (13.71%) | 0 (0.00%) | 0 (0.00%) | 7196 (20.18%) |
| 2013 | 32102 | 16910 (52.68%) | 3394 (10.57%) | 0 (0.00%) | 0 (0.00%) | 11798 (36.75%) |
| 2014 | 32355 | 484 (1.50%) | 23 (0.07%) | 0 (0.00%) | 0 (0.00%) | 31848 (98.43%) |
| 2015 | 30414 | 0 (0.00%) | 0 (0.00%) | 0 (0.00%) | 0 (0.00%) | 30414 (100.00%) |
| 2016 | 29469 | 0 (0.00%) | 0 (0.00%) | 0 (0.00%) | 0 (0.00%) | 29469 (100.00%) |
| 2017 | 29491 | 1 (0.00%) | 0 (0.00%) | 0 (0.00%) | 0 (0.00%) | 29490 (100.00%) |
| 2018 | 28746 | 0 (0.00%) | 0 (0.00%) | 0 (0.00%) | 0 (0.00%) | 28746 (100.00%) |
| 2019 | 27689 | 0 (0.00%) | 0 (0.00%) | 0 (0.00%) | 0 (0.00%) | 27689 (100.00%) |
| 2020 | 25155 | 0 (0.00%) | 0 (0.00%) | 0 (0.00%) | 0 (0.00%) | 25155 (100.00%) |
| 2021 | 24680 | 0 (0.00%) | 0 (0.00%) | 0 (0.00%) | 0 (0.00%) | 24680 (100.00%) |
| 2022 | 22740 | 0 (0.00%) | 0 (0.00%) | 0 (0.00%) | 0 (0.00%) | 22740 (100.00%) |
| 2023 | 23596 | 0 (0.00%) | 0 (0.00%) | 0 (0.00%) | 0 (0.00%) | 23596 (100.00%) |

Incomplete indicates a non-missing geographic code that could not be resolved to the county level. County-level comparison was considered available when both the household registration and current residence codes could be resolved to the county level.

Table S3. Agreement between household registration type and address-based classification among notified tuberculosis cases, 2014-2023.

| Household Registration Type | Address-based classification | | Total |
| --- | --- | --- | --- |
|  | Concordant | Migratory |  |
| Local | 160,965 | 1,043 | 162,008 |
| Migrant | 35,219 | 75,953 | 111,172 |
| Total | 196,184 | 76,996 | 273,180 |

Overall agreement = 86.73%; Cohen’s κ = 0.711. For this analysis, household registration type was dichotomized as Local versus Migrant, with Migrant including intracity, intraprovincial, and interprovincial mobility; HKMT and Foreign cases were excluded. Under the address-based classification, cases whose household registration and current residence codes differed at the county level were classified as migratory, while cases with concordant codes were classified as concordant. HKMT, Hong Kong, Macao, and Taiwan.

Table S4. Model Specifications and Diagnostics for the Age-Period-Cohort Analyses.

| Analysis | Analysis period | Age range | Population | Age groups, n | Periods, n | Cohorts, n | Residual df | Deviance/df | Pearson dispersion |
| --- | --- | --- | --- | --- | --- | --- | --- | --- | --- |
| Main analysis | 2009-2023 | 0-94 years | Overall | 19 | 3 | 21 | 17 | 27.38 | 27.48 |
| Main analysis | 2009-2023 | 0-94 years | Male | 19 | 3 | 21 | 17 | 28.11 | 28.32 |
| Main analysis | 2009-2023 | 0-94 years | Female | 19 | 3 | 21 | 17 | 5.89 | 5.85 |
| Sensitivity analysis | 2009-2023 | 0-84 years | Overall | 17 | 3 | 19 | 15 | 30.91 | 31.03 |
| Sensitivity analysis | 2009-2023 | 0-84 years | Male | 17 | 3 | 19 | 15 | 31.55 | 31.79 |
| Sensitivity analysis | 2009-2023 | 0-84 years | Female | 17 | 3 | 19 | 15 | 6.61 | 6.56 |
| Sensitivity analysis | 2005-2019 | 0-94 years | Overall | 19 | 3 | 21 | 17 | 80.98 | 80.50 |
| Sensitivity analysis | 2005-2019 | 0-94 years | Male | 19 | 3 | 21 | 17 | 62.74 | 62.33 |
| Sensitivity analysis | 2005-2019 | 0-94 years | Female | 19 | 3 | 21 | 17 | 19.52 | 19.42 |

df, degrees of freedom. Deviance/df was calculated as residual deviance divided by the residual degrees of freedom. Pearson dispersion was calculated as Pearson χ² divided by the residual degrees of freedom, with values >1 indicating overdispersion. All models converged. Reported 95% CIs were based on Pearson-scaled standard errors. CI, confidence interval.

Table S5. Global Moran's I for County-Level Reported Tuberculosis Incidence and the Proportions of Migrant and Local Tuberculosis Cases in Zhejiang Province, 2005-2023.

| Indicator | Period | Moran's I | Z score | P value |
| --- | --- | --- | --- | --- |
| Reported TB incidence | 2005-2009 | 0.034 | 0.683 | 0.495 |
| Reported TB incidence | 2010-2014 | 0.17 | 2.41 | 0.0161 |
| Reported TB incidence | 2015-2019 | 0.341 | 4.67 | <0.0001 |
| Reported TB incidence | 2020-2023 | 0.387 | 5.3 | <0.0001 |
| Proportion of migrant TB cases | 2005-2009 | 0.483 | 6.52 | <0.0001 |
| Proportion of migrant TB cases | 2010-2014 | 0.533 | 7.16 | <0.0001 |
| Proportion of migrant TB cases | 2015-2019 | 0.549 | 7.39 | <0.0001 |
| Proportion of migrant TB cases | 2020-2023 | 0.576 | 7.74 | <0.0001 |
| Proportion of local TB cases | 2005-2009 | 0.483 | 6.52 | <0.0001 |
| Proportion of local TB cases | 2010-2014 | 0.533 | 7.16 | <0.0001 |
| Proportion of local TB cases | 2015-2019 | 0.549 | 7.39 | <0.0001 |
| Proportion of local TB cases | 2020-2023 | 0.576 | 7.74 | <0.0001 |

Z scores and P values were obtained from the analytical Moran's I test under the randomization assumption. Moran's I values were identical for migrant and local TB case proportions because the two proportions were complementary within each spatial unit. TB, tuberculosis.

Table S6. Rate Stability and Empirical Bayes Sensitivity Analysis of County-Level Reported Tuberculosis Incidence in Zhejiang Province, 2005-2023.

| Period | Minimum incident cases | Maximum RSE (%) | Median absolute rate change  (per 100,000) | Raw Moran’s I | EB Moran’s I | Raw ≥95% Gi* clusters | EB ≥95% Gi* clusters |
| --- | --- | --- | --- | --- | --- | --- | --- |
| 2005-2009 | 146 | 8.28 | 0.0414 | 0.0342 | 0.034 | 0 | 0 |
| 2010-2014 | 95 | 10.3 | 0.0714 | 0.17 | 0.17 | 7 | 7 |
| 2015-2019 | 56 | 13.4 | 0.0798 | 0.341 | 0.34 | 12 | 12 |
| 2020-2023 | 45 | 14.9 | 0.0946 | 0.387 | 0.384 | 13 | 13 |

RSE, relative standard error; EB, empirical Bayes; TB, tuberculosis. Gi* ≥95% counts include both hot and cold spots.

Table S7. Distribution of interprovincial migratory tuberculosis cases by household-registration province, Zhejiang Province, 2014-2023.

| Household-registration province | No. of cases | Proportion (%) |
| --- | --- | --- |
| Guizhou | 16,832 | 24.8 |
| Sichuan | 7,723 | 11.4 |
| Anhui | 7,105 | 10.5 |
| Yunnan | 6,602 | 9.7 |
| Jiangxi | 6,536 | 9.6 |
| Hubei | 4,328 | 6.4 |
| Hunan | 4,256 | 6.3 |
| Henan | 3,838 | 5.7 |
| Chongqing | 3,510 | 5.2 |
| Shaanxi | 1,111 | 1.6 |
| Jiangsu | 883 | 1.3 |
| Heilongjiang | 778 | 1.1 |
| Guangxi | 720 | 1.1 |
| Fujian | 618 | 0.9 |
| Shandong | 610 | 0.9 |
| Gansu | 547 | 0.8 |
| Jilin | 384 | 0.6 |
| Hebei | 258 | 0.4 |
| Guangdong | 252 | 0.4 |
| Liaoning | 234 | 0.3 |
| Shanxi | 213 | 0.3 |
| Xinjiang | 160 | 0.2 |
| Inner Mongolia | 122 | 0.2 |
| Qinghai | 86 | 0.1 |
| Ningxia | 49 | 0.1 |
| Hainan | 47 | 0.1 |
| Shanghai | 42 | 0.1 |
| Beijing | 24 | <0.1 |
| Tianjin | 24 | <0.1 |
| Tibet | 24 | <0.1 |
| Total | 67,916 | 100.0 |

Household-registration province was treated as the origin province. Proportions were calculated using all interprovincial migratory TB cases with current residence in Zhejiang Province during 2014-2023 as the denominator. TB, tuberculosis.

Table S8. Model Performance in the Primary Analysis and Temporal and Geographic Validation of the Overall Boosted Regression Tree Model, Zhejiang Province, 2014-2023.

| Measure | Primary analysis | Temporal validation | Geographic validation |
| --- | --- | --- | --- |
| Analysis design | 50 repeated stratified subsamples with 70:30 training-test splits | 2014-2019 training; 2020-2023 testing | 5-fold whole-origin-city holdout |
| Training migratory cases, n | 5,244 | 51,423 | 60,385 (54,996-64,162) |
| Training comparison cases, n | 13,430 | 121,175 | 156,803 (109,048-173,923) |
| Testing migratory cases, n | 2,253 | 23,594 | 14,632 (10,855-20,021) |
| Testing comparison cases, n | 5,760 | 70,770 | 35,142 (18,022-82,897) |
| Testing AUC | 0.967 (0.961-0.971) | 0.924 | 0.841 (0.740-0.969) |
| GRP per capita | 57.5 (54.5-59.5) | 64.9 | 53.5 (49.9-69.1) |
| Tertiary industry proportion | 5.9 (4.7-7.4) | 8.9 | 7.1 (2.3-9.1) |
| Emigration index | 4.3 (3.5-5.5) | 3.7 | 5.3 (3.3-14.2) |
| Immigration index | 9.0 (7.3-12.3) | 3.0 | 8.5 (1.0-24.7) |
| Population density | 4.2 (3.0-5.4) | 3.5 | 2.3 (0.9-12.2) |
| City type | 6.8 (6.0-7.8) | 8.4 | 8.1 (2.6-16.2) |
| Age | 3.3 (2.8-3.9) | 2.3 | 2.7 (1.1-3.8) |
| Occupation | 4.5 (3.7-5.3) | 4.4 | 3.9 (1.9-5.2) |
| Hospital beds | 3.5 (2.9-4.5) |  | 3.4 (0.2-3.7) |
| Secondary industry proportion |  |  |  |
| Sex |  |  |  |

For the primary analysis, testing AUCs and relative contributions are reported as medians with 95% intervals across 50 repeated analyses. Geographic-validation values are reported as medians with ranges across five folds. Temporal-validation values are from a single temporal split. Predictor rows report relative contributions (%). Values <2% are omitted. AUC, area under the receiver operating characteristic curve; GRP, gross regional product.

Table S9. Sensitivity Analyses of the Overall Boosted Regression Tree Model, Zhejiang Province, 2014-2023.

| Measure | Primary analysis | Restrictive definition | 2019 only | No AMAP | Pre-COVID-19 |
| --- | --- | --- | --- | --- | --- |
| Study period | 2014-2023 | 2014-2023 | 2019 | 2014-2023 | 2014-2019 |
| Analysis specification | Address-based classification | Agreement between classifications in both groups | Restricted to 2019 | AMAP mobility indices excluded | Restricted to 2014-2019 |
| Eligible migratory TB cases, n | 75,017 | 74,026 | 7,261 | 75,017 | 51,423 |
| Eligible comparison cases, n | 191,945 | 158,473 | 19,594 | 191,945 | 121,175 |
| Testing AUC, median (empirical 95% interval) | 0.967  (0.961-0.971) | 0.972  (0.967-0.977) | 0.956  (0.933-0.973) | 0.966  (0.960-0.970) | 0.972  (0.967-0.975) |
| GRP per capita | 57.5  (54.5-59.5) | 57.8  (55.8-59.7) | 76.1  (71.8-81.1) | 62.1  (60.5-64.1) | 63.9  (61.8-65.7) |
| Tertiary industry proportion | 5.9  (4.7-7.4) | 6.1  (4.8-7.4) | 2.8  (0.6-5.2) | 8.2  (7.0-9.6) | 8.7  (7.4-10.1) |
| Emigration index | 4.3  (3.5-5.5) | 3.3  (2.6-4.2) | 0.8  (0.3-1.8) | Not included | 2.7  (1.7-4.4) |
| Immigration index | 9.0  (7.3-12.3) | 8.2  (6.0-10.3) | 1.3  (0.7-2.6) | Not included | 3.4  (2.5-4.3) |
| Population density | 4.2  (3.0-5.4) | 4.6  (3.7-5.7) |  | 6.2  (5.1-8.0) | 3.7  (2.8-4.5) |
| City type | 6.8  (6.0-7.8) | 6.9  (5.8-7.7) | 7.1  (4.9-9.3) | 8.9  (8.0-9.9) | 8.4  (7.1-9.7) |
| Age | 3.3  (2.8-3.9) | 4.0  (3.4-4.8) | 2.1  (1.3-3.3) | 3.3  (2.7-3.8) | 2.6  (2.1-3.3) |
| Occupation | 4.5  (3.7-5.3) | 4.8  (4.2-5.6) | 4.1  (3.0-6.5) | 5.2  (4.3-6.1) | 4.9  (3.9-6.5) |
| Hospital beds | 3.5  (2.9-4.5) | 3.7  (2.9-4.3) | 2.8  (0.5-5.3) | 4.7  (4.1-5.6) |  |
| Secondary industry proportion |  |  |  |  |  |
| Sex |  |  |  |  |  |

Testing AUCs and relative contributions are reported as medians with 95% intervals across 50 repeated analyses. The restrictive definition required agreement between household registration type and the address-based classification in both groups. AMAP indices were excluded in the no-AMAP analysis; values <2% are omitted except for the AMAP indices in the 2019-only analysis. AUC, area under the receiver operating characteristic curve; AMAP, AutoNavi Map; GRP, gross regional product.

2 **Supplementary Figures**

Figure S1. Joinpoint Trends in Reported Tuberculosis Incidence in Zhejiang Province, 2005-2023.

Figure S2. Joinpoint Trends in the Proportion of Migrant Tuberculosis Cases in Zhejiang Province, 2005-2023.

Figure S3. Observed Age-Specific Reported Tuberculosis Incidence by Calendar Period in Zhejiang Province, 2009-2023. (A) Overall population. (B) Males. (C) Females. Observed age-specific reported TB incidence is shown for the three periods 2009-2013, 2014-2018, and 2019-2023.

Figure S4. Sensitivity Analyses of Age, Period, and Cohort Effects on Reported Tuberculosis Incidence in Zhejiang Province. (A-C) Primary analysis, 2009-2023, ages 0-94 years. (D-F) Age-range sensitivity analysis, 2009-2023, ages 0-84 years. (G-I) Alternative-period sensitivity analysis, 2005-2019, ages 0-94 years. Shaded areas indicate 95% CIs. CI, confidence interval.

Figure S5. Temporal Patterns of County-Level Getis-Ord Gi* Hot and Cold Spots in the Proportions of Migrant and Local Tuberculosis Cases in Zhejiang Province, 2005-2023. (A) Hot and cold spots in the proportion of migrant TB cases and (B) hot and cold spots in the proportion of local TB cases for 2005-2009, 2010-2014, 2015-2019, and 2020-2023. Maps display the 90%, 95%, and 99% confidence levels; the 90% level was considered exploratory. TB, tuberculosis.

Figure S6. False Discovery Rate-Adjusted Sensitivity Analysis of County-Level Getis-Ord Gi* Hot and Cold Spots in Zhejiang Province, 2005-2023. (A) Reported TB incidence, (B) migrant TB case proportion, and (C) local TB case proportion for 2005-2009, 2010-2014, 2015-2019, and 2020-2023. Benjamini-Hochberg FDR correction was applied to the local Getis-Ord Gi* tests. TB, tuberculosis; FDR, false discovery rate.
